# Reduced withdrawal and failure rates of accelerated nursing students enrolled in pharmacology is associated with a supportive intervention

**DOI:** 10.1186/s12909-016-0570-z

**Published:** 2016-02-01

**Authors:** Sheila Anne Doggrell, Sally Schaffer

**Affiliations:** School of Biomedical Sciences, Faculty of Health, Queensland University of Technology, Brisbane, GPO 2343, QLD4001 Australia

**Keywords:** Accelerated nursing students, Domestic graduate, Domestic students with non-university qualifications, International students, Retention, Pharmacology, Withdrawal

## Abstract

**Background:**

To reduce nursing shortages, accelerated nursing programs are available for domestic and international students. However, the withdrawal and failure rates from these programs may be different than for the traditional programs. The main aim of our study was to improve the retention and experience of accelerated nursing students.

**Methods:**

The academic background, age, withdrawal and failure rates of the accelerated and traditional students were determined. Data from 2009 and 2010 were collected prior to intervention. In an attempt to reduce the withdrawal of accelerated students, we set up an intervention, which was available to all students. The assessment of the intervention was a pre-post-test design with non-equivalent groups (the traditional and the accelerated students). The elements of the intervention were a) a formative website activity of some basic concepts in anatomy, physiology and pharmacology, b) a workshop addressing study skills and online resources, and c) resource lectures in anatomy/physiology and microbiology. The formative website and workshop was evaluated using questionnaires.

**Results:**

The accelerated nursing students were five years older than the traditional students (p < 0.0001). The withdrawal rates from a pharmacology course are higher for accelerated nursing students, than for traditional students who have undertaken first year courses in anatomy and physiology (p = 0.04 in 2010). The withdrawing students were predominantly the domestic students with non-university qualifications or equivalent experience. The failure rates were also higher for this group, compared to the traditional students (p = 0.05 in 2009 and 0.03 in 2010). In contrast, the withdrawal rates for the international and domestic graduate accelerated students were very low. After the intervention, the withdrawal and failure rates in pharmacology for domestic accelerated students with non-university qualifications were not significantly different than those of traditional students.

**Conclusions:**

The accelerated international and domestic graduate nursing students have low withdrawal rates and high success rates in a pharmacology course. However, domestic students with non-university qualifications have higher withdrawal and failure rates than other nursing students and may be underprepared for university study in pharmacology in nursing programs. The introduction of an intervention was associated with reduced withdrawal and failure rates for these students in the pharmacology course.

## Background

Ongoing nursing shortages in many countries have driven the need to train more nurses [1–4]. Additionally, it is widely acknowledged by health policy makers, providers, clinicians, and social scientists, that a diverse healthcare workforce will improve health disparities in ethnic and other socially disadvantaged groups (reviewed in [5]). Thus, in many countries, universities face the challenge of graduating increasing numbers of students and diversifying the population of nurses.

One of the universal primary strategies for increasing participation in nursing education, including in Australia, has been the introduction of accelerated nursing programs. Students, entering these programs, are granted academic credit for prior learning in a related, or an unrelated, field or for equivalent life or workplace experience. These accelerated Bachelor of Nursing programs, provide many advantages, including a shortened study time and a concomitant reduction in university expenses.

In Australia, accelerated students are a mix of graduate and non-graduate students. The graduate students are either international or domestic students with a degree from a university program other than nursing. The non-graduates include those with non-university qualifications, predominantly with a nursing diploma from a college of technical and further education (TAFE), and those with equivalent life or work experience.

There are limited studies as to whether withdrawal and/or failure rates are different in accelerated than traditional nursing programs. The accelerated students can be graduates (domestic or international). Two studies have shown that non-nursing domestic graduates completing accelerated nursing programs, including in Australia, do as well or better than students completing traditional nursing programs (reviewed in [6, 7]). In contrast, there is only one recent study, of international graduate students, and this study suggests that international graduate students are also as successful as domestic students, although this study was limited by the small numbers [8]. In Australia, TAFE colleges offer vocational education and training for nurses and provide ‘a pathway to university education where diploma graduates can receive up to one or two year’s credit towards a related university degree’ [9]. However, to our knowledge, there is no literature relating to withdrawal, failure or success rates of diploma nurses undertaking accelerated internal nursing programs.

At our Australian university, we offer a three year undergraduate Bachelor of Nursing degree, as well as a two year accelerated program which allows students granted academic credit, to enter this course at second year level. The accelerated students complete a second year level pharmacology course, in their first semester at university, in a class with traditional students who are in their second year of study. As a consequence of entering at second year level, the accelerated students miss out on the first year courses including anatomy, physiology and microbiology.

At our university, in 2009 and 2010, the withdrawal rates for accelerated students in the pharmacology course were higher than for traditional students. These students were withdrawing from the nursing course and leaving the university. This prompted us to consider ways of improving the retention of accelerated students. Previously, there has been no research into interventions to improve the retention of accelerated nursing programs for graduates [10] or, to our knowledge, for non-graduates.

We determined the academic background, age and withdrawal rates of the accelerated and traditional student and this showed that the withdrawal rates were higher for the accelerated than traditional students. Further analysis indicated that withdrawal rates were highest among the non-graduates (TAFE) students. Subsequently, we did a search for an intervention model that may be useful for our accelerated students. A model proposed by Yorke & Thomas (2003) found a number of factors had a positive impact on the retention of students from low socioeconomic backgrounds and their subsequent success at university [11]. It seemed to us, this model could be useful, as students from TAFE in Australia have, on average, a lower socioeconomic status than those attending university [12, 13]. The factors identified by Yorke & Thomas (2003) included: provision of a helpful institutional environment, provision of support during the first year of study and introduction of early formative assessment [11]. These factors formed the basis of the intervention we used.

The main aim of this study was to determine whether an intervention strategy, based on the factors identified by Yorke & Thomas (2003) [11], could support a diverse cohort of accelerated students in the pharmacology course. We determined the success of the intervention by measuring withdrawal rates, marks and failure rates of accelerated students before and after the intervention, compared with traditional students, and obtained student feedback regarding the intervention through completion of questionnaires. Our results show the intervention was associated with a reduction in the withdrawal and failure rates of the non-graduates.

## Methods

### Ethics

The project was submitted to our Institutional Research Ethics committee for consideration in 2009. The ethics committee communicated that the project did not require further clearance, provided it was undertaken in accordance with the (Australian) National Statement on Ethical Conduct in Human Research, which it was. For instance, this includes students having the choice whether or not to respond to questionnaires, and an undertaking that this will not affect their marks. Students are not required to give any information that will identify them in questionnaires. By choosing to answer the questionnaire, students are deemed to have given consent.

### Academic background, gender and age of accelerated students

Students were not identified by name, but by student ID number. The authors identified the traditional and accelerated students by obtaining course class lists through an academic and administrative web portal, the Student and Academic Management System (SAMS). We identified the accelerated students as those who were given academic credit for previous educational experience (advanced standing) by the university, and were completing a two year degree. The gender and date of birth of each student was available, and the age of each student at the start of the year was calculated from the date of birth. The academic background (previous secondary or tertiary qualifications) of accelerated students was also determined.

The accelerated students were divided into three groups. The first group were international graduate students, who were identified as non-Australian citizens (foreign born and/or educated) and categorised as ‘international’ for the purposes of paying fees. The other two groups were domestic students, who were defined as having Australian citizenship and receiving commonwealth support for university fees: one of these groups were domestic university graduates who had graduated from a university program other than nursing; the third group were domestic non-graduates with non-university qualifications (e.g. diploma) or no tertiary qualifications but equivalent work experience. The other students in the cohort were identified as traditional students, who were completing the traditional three year degree and served as a control group.

We also used SAMS, to determine the attrition rates for all students. The final course marks were noted for students who completed the course.

### Background to intervention

Of particular interest to us, in developing an intervention was a study that was selective for students from lower SES backgrounds. Yorke & Thomas (2003) identified six Universities in the UK who were performing above the average for completion rates for one of the following groups: young entrants from working-class backgrounds, young entrants from neighbourhoods with low participation rates, and mature entrants with no familial experience of high education and from low participation neighbourhoods [11]. These low SES backgrounds are probably similar to those of our non-university graduates with diplomas from TAFE [12, 13]. After identification, Yorke & Thomas (2003) questioned these institutions, who were doing well with low SES students, about what they were doing that might account for better completion rates than the benchmark [11]. The study concluded that the following factors have a positive impact on the retention of these students and their subsequent success [11]:

1. An institutional climate supportive in various ways of students’ development i.e. perceived as ‘friendly’. For instance, students are more likely to persist at university, if they have developed a relationship with the Institution and consider the Institution will help them realise their goals.
2. An emphasis on support leading up to, and during, the critically important first year of study.
3. An emphasis on formative assessment in the early phase of programmes, and this assessment should have feedback from the teacher [14].
4. Recognition of the importance of the social dimension in learning activities. This can include group learning.
5. Recognition that the pattern of students’ engagement in higher education was changing, and a preparedness to respond positively to this in various ways. This may take the form of staff development activities to facilitate change in teaching and learning practices in support of the needs of a more diverse student cohort.

Kuh et al (2007) reported the need to verify effective approaches that foster success of different groups of students at different types of institution [15]. As far as we are aware, there are no reports of any attempt to use the findings of Yorke & Thomas (2003) [11] to design an intervention to increase the retention of low SES students. Since our research was aimed at reducing the withdrawal and improving the success of accelerated students, some of whom came from similar backgrounds as those identified in the York and Thomas (2003) study, we decided to base our intervention on factors one to four of the Yorke & Thomas (2003) model. The fifth factor is a staff development issue, and not within the scope of the intervention.

In our intervention, the provision of a one day Workshop held in “O-week” (orientation week) allowed students to engage and form a relationship with staff at an early stage. The resource lectures and the formative website activity helped students come to terms with the expectation of the university, without leaving students struggling with the burden of ‘failing and trailing’ [11]. Activities such as these can support students leading up to commencement of their studies. The “O week” Workshop provided opportunities for social learning during the interactive active learning activities.

The assessment of the study was pre-post-test design with non-equivalent groups (the traditional and the accelerated students). The data from 2009 and 2010 were collected prior to the intervention, and from 2011 and 2012 with the intervention.

### The intervention

For equity reasons, the intervention was available to all the students who were enrolled in the pharmacology course in February 2011 and 2012, enabling access to the “Bioscience and Pharmacology for Advanced Standing Students” community website on Blackboard. The students were advised by email prior to the start of semester, of the website details and invited to access the support material as well as to attend the workshop during “O Week”. The students were also advised during a face-to-face pharmacology lecture in the first week of semester, of the availability of the community website, with all the components of the intervention. Thus, the intervention was available to both the accelerated and traditional students.

The intervention included a community website providing printable and recorded material in four folders: (1) “Information”, (2) “Getting Started”, (3) “O Week Workshop”, (4) “Resource Lectures”. The “O Week Workshop” and “Resource Lectures” were delivered face-to-face; lecture notes were also made available. The “Information” folder contains an introduction to and explanation of the activities available on the website and face-to-face.

In order to be able to study pharmacology, students need a good background in anatomy, physiology and microbiology. The traditional students are provided with this knowledge in their first year of study, but the accelerated students miss out on this, at our university. Thus, the first part of our intervention, the “Getting Started” folder, includes eChapters on introductory anatomy, physiology and pharmacology concepts: medical and anatomical terminology, cell to tissues, tissues to body, homeostasis, physiological feedback mechanisms, binding sites – the key to pharmacology, physiological processes – links to pharmacokinetics. Following each eChapter, there was a formative activity in the form of on-line Multiple Choice Question (MCQ) quizzes, with feedback for each correct or incorrect response entered by the students.

All nursing students enrolled in the pharmacology course were invited to the second part of the intervention; a three hour workshop in “O week” prior to semester 1. The workshop presentations started with an introductory welcome to the intervention, which included a discussion of why the intervention was set up i.e. data showing high withdrawal rates in previous accelerated student cohorts. The introduction also included a demonstration of how to access and use the community website and the Blackboard sites for the pharmacology course. This was followed by a demonstration of library resources available on the library website (e.g. chat to a librarian, QUT cite/write, searching databases), and within the library (e.g. workshops on writing academic essay, developing effective presentation skills) given by a QUT Health Sciences librarian. Next, there was a discussion regarding effective learning strategies including different learning styles, active learning, and time management strategies enabling success at university. Finally, a previous accelerated nursing student discussed how he/she “survived” the pharmacology course, and this included their first impressions of the university/course, how they approached the workload, how they managed study and other commitments (e.g. family), and how they succeeded in the courses. All of the presentations were recorded, and added to the “O Week Workshop” folder on the community website.

All nursing students enrolled in the pharmacology course were also invited to the third part of the intervention; the “Resource Lectures” were face-to-face lectures of two hours of introductory anatomy and physiology material covering the nervous, endocrine, cardiovascular, respiratory, digestive and renal systems. A two hour introductory microbiology lecture discussed the diversity of microorganisms in relation to human health, the structures of these microorganisms and an introduction to diagnosis of infections. The notes and recordings of these lectures were also posted on the community website.

### Evaluation of “O Week Workshop” and “Resource Lectures”

At the end of the workshop and resource lectures, the students present were asked to complete an anonymous questionnaire to evaluate the presentations and lectures. The students responded to each statement using a 5-point Likert scale, with responses ranging from ‘strongly agree’ to ‘strongly disagree’. The statements were:

- The information I have received prior to this week has been timely and informative
- These orientation sessions were scheduled at a suitable time
- The facilities and location were appropriate and satisfactory
- The Library information session was informative and valuable
- The Active Learning session was informative and valuable
- The session by the previous Advanced Standing student was informative and valuable
- The review of Anatomy and Physiology was informative and valuable
- The review of Microbiology was informative and valuable

The students were also asked the following open-ended questions:

- In what ways could this orientation have been improved to better suit your needs?
- Would you recommend this session to other students?
- Please suggest other topics and/or support services that would be useful to advanced standing students.
- Any other comments?

Students, who completed the questionnaire, entered a raffle to win either a Bioscience or Pharmacology textbook. The questionnaires were subsequently analysed by the authors.

### Questionnaire on “Getting Started”

An open-ended questionnaire was sent by email to all students in the pharmacology course in week 11 of the semester and after their end-of-semester exam in 2011 (end-of-semester questionnaire).

The students were asked to submit an anonymous response to a third party, who was not a teacher on the course, on the following:

*The Getting Started section of the Community website contained eChapters on key topics in bioscience and pharmacology, including Medical terminology and Pharmacodynamics, the Keys to Pharmacology, followed by quizzes for self-testing.*

*Did you use any of these?*

*О Yes (please comment below)О No (please comment below)*

*Aspects you could comment on: What was the best and worst thing about this section on the website? How could the website be improved? Are there any other concepts that you would like to have covered? If you did not use the Getting started section, why not? On reflection, do you think it would have been helpful to use this website before or at the beginning of the semester?*

The response rate was very low (ten responses) in 2011, which is standard at our university for an end-of-semester email survey. Thus, an end-of-semester questionnaire was not sent to students in 2012.

### Analysis of data

A comparison of the age and gender of traditional and accelerated students was made using Students unpaired *t*-test. Comparisons between the age and gender of sub-groups of accelerated students used ANOVA, and if significance was found, post-hoc analysis was by Students unpaired *t*-test. *T*-test and ANOVA data were determined using Excel 10.

Comparisons between withdrawal, failure and pass rates for different groups of students were made by determining the Odds ratio with 95 % confidence levels (OR with 95 % CI) using the MedCalc statistical calculator: https://www.medcalc.org/calc/odds_ratio.php. For all statistics used, a p value of < 0.05 was considered significant.

The responses to the Likert scale part of the questionnaire on the “O Week Workshop” and “Resource Lectures” were calculated as percentages; the responses were treated as ordinal data, thus no further statistical analysis was completed. The comment responses from the “O Week Workshop” and “Getting Started” were tabulated. As there were low numbers of comment responses to the questionnaires, we did not undertake any further analysis of this.

The numbers of students, accessing the community website in 2011 and 2012 was noted; student names were not identified.

## Results

### Age and gender of students

Tables 1 and 2 report the age and gender for the traditional and accelerated nursing students, and subgroups of accelerated students. The mean age of the accelerated students was significantly greater by about 5 years than the traditional students (Table 1). Of the subgroups of accelerated students, the domestic graduate students were older than the international graduate students, and for some of the years this reached significance (Table 2). In 2009, the domestic non-graduates were younger than the domestic graduates, but in 2010-2012, the ages of the domestic non-graduates and graduates were not significantly different (Table 2). For each year, the students were predominantly female (Tables 1 and 2).

**Table 1:** Comparison of the age and gender of the traditional and accelerated students in the pharmacology course

	Age (years)			Gender %F/%M	
Year	Traditional	Accelerated	*p*-value	Traditional	Accelerated
2009	22.4 ± 0.4 (n = 246)	28.8 ± 0.8 (n = 146)	<0.0001	89/11	84/16
2010	25.6 ± 0.5 (n = 296)	29.8 ± 1.0 (n = 105)	<0.0001	85/15	78/22
2011	23.7 ± 0.5 (n = 254)	28.5 ± 0.5 (n = 197)	<0.0001	85/15	82/18
2012	25.4 ± 0.5 (n = 229)	29.0 ± 0.4 (n = 319)	<0.0001	90/10	84/16

*F* female, *M* male

*p*-value is for Students unpaired *t*-test for age of traditional and accelerated students

**Table 2:** Comparison of the age and gender of accelerated students sub-groups in the pharmacology course

	Age (years)		*p*-value *		*p*-value **
Year	International	Domestic graduates		Domestic non-graduates	
2009	25.7 ± 0.8 (51)	32.9 ± 2.2 (33)	p = 0.003	28.1 ± 1.3 (62)	p = 0.04
2010	27.5 ± 1.0 (39)	31.7 ± 3.0 (23)	p = 0.11	31.6 ± 1.6 (43)	p = 0.97
2011	27.5 ± 0.5 (93)	29.1 ± 1.1 (42)	p = 0.13	28.9 ± 0.9 (86)	p = 0.9
2012	28.0 ± 0.4 (135)	31.6 ± 1.3 (49)	p = 0.001	29.2 ± 0.8 (122)	p = 0.1
	Gender %F/%M				
Year	International	Domestic graduates		Domestic non-graduates	
2009	78/22	86/14		83/17	
2010	55/45	80/20		84/16	
2011	77/23	88/13		80/20	
2012	83/17	78/22		85/15	

*p value between international and domestic graduate accelerated students

**p value between domestic graduate and domestic TAFE students

*F* female, *M* male

### Before the intervention; student withdrawal and failure rates

Before the intervention (2009 and 2010) the withdrawal rates for accelerated students were 7 % (10 of 146 students) and 8.3 % (9 of 105) respectively, and these rates were significantly higher than for the traditional students who had completed first year in 2010 but not in 2009 (Table 3). Further analysis of the accelerated students showed that the withdrawal rates of international students and domestic graduate students were low in these years (Table 4). However, the domestic students with non-university qualifications or equivalent experience had high withdrawing rates; 11.9 % in 2009 and 18.6 % in 2010, and these rates were significantly higher than for the traditional students (Table 3).

**Table 3:** Odds ratios for withdrawal and failure rates of traditional versus accelerated and domestic non-university graduates before (2009-2010) and after (2011-2012) the intervention

Withdrawal rates before (2009-2010) and after (2011-2012) the intervention				
Year	Groups	Values	OR (95 % CI)	*p*-Value
2009	Traditional vs accelerated	4 vs 7 %	1.81 (0.52 to 6.4)	p = 0.35
	Traditional vs domestic (non-uni)	4 vs 11.9 %	0.30 (0.095 to 0.983)	p = 0.05
2010	Traditional vs accelerated	1 vs 8.3 %	8.61 (1.05 to 70.17)	p = 0.04
	Traditional vs domestic (non-uni)	1 vs 18.8 %	0.04 (0.0056 to 0.3286)	p = 0.002
2011	Traditional vs accelerated	4 vs 2 %	0.51 (0.091 to 2.852)	p = 0.49
	Traditional vs domestic (non-uni)	4 vs 4.6 %	0.79 (0.20 to 3.04)	p = 1.26
2012	Traditional vs accelerated	1.5 vs 1 %	0.49 (0.044 to 5.55)	p = 0.56
	Traditional vs domestic (non-uni)	1.5 vs 3.3 %	1.51 (0.25 to 9.27)	p = 1.00
Failure rates before (2009-2010) and after (2011-2012) the intervention				
2009	Traditional vs domestic (non-uni)	9 vs 19.4 %	2.37 (1.02 to 5.53)	p = 0.05
2010	Traditional vs domestic (non-uni)	6 vs 16.3 %	2.98 (1.12 to 7.98)	p = 0.03
2011	Traditional vs domestic (non-uni)	2 vs 5.8 %	3.13 (0.62 to 15.89)	p = 0.17
2012	Traditional vs domestic (non-uni)	4 vs 6.7 %	0.85 (0.28 to 2.62)	_p = 0.35

**Table 4:** Withdrawal and failure rates of subgoups of accelerated nursing students before (2009-2010) and after (2011-2012) the intervention

Year	International students			Domestic University graduates			Domestic non-university qualifications or equivalent experience		
	Number	Withdrew (%)	Failed (%)	Number	Withdrew (%)	Failed (%)	Number	Withdrew (%)	Failed (%)
2009	51	0 %	3.9 %	33	0 %	3.0 %	62	11.9 %	19.4 %
2010	39	0 %	0 %	23	0 %	8.7 %	43	18.6 %	16.3 %
2011	93	0 %	0 %	42	2.3 %	0 %	86	4.6 %	5.8 %
2012	135	0 %	0 %	49	2.0 %	0 %	122	3.3 %	6.7 %

Before the intervention, the failure rates of the combined cohort of accelerated students (10 %) were not significantly different from the failure rates of the traditional students, 6-9 %. However, subgroup analysis showed higher failure rates for the non-graduate accelerated students, than the traditional students in 2009 and 2010 (Table 3).

### Feedback on “Getting started” part of the intervention

In 2011, only ten accelerated students responded to the end-of-semester questionnaire on “Getting started” with their comments and reflections. Those students who used “Getting Started” found it very useful, whether they had prior anatomy and physiology knowledge or not (Table 5).

**Table 5:** Comments on “Getting started” and “Workshop”

“Getting Started”	“Workshop”
*Aspects you could comment on: What was the best and worst thing about this section on the website? How could the website be improved? Are there any other concepts that you would like to have covered? If you did not use the Getting started section, why not? On reflection, do you think it would have been helpful to use this website before or at the beginning of the semester?*	*Any other comments*: *Positive comments*
I used the “Getting Started” activities and I found everything very useful. I finished TAFE 6 months before starting uni and these activities were refreshing my knowledge. In my opinion the website was easy to use.	I found everything that was covered was quite beneficial
I thought the Getting Started Program was great, helping in refreshing my knowledge, I would recommend that every advanced standing student should access this before they start their lectures. The content I feel covered most at an introduction stage though may need to look at disease state a little more for medication wise	A great help
I printed off the eBook on medical and anatomical terminology and throughout the semester I did refer back to it to help me learn some new words. That part was great. I didn’t get a chance to do the online test as I wasn’t aware of it until later in the semester and by the time you do all the tuts, other online test I just out of time to fit them in. However as I find the online test in other areas such Pharmacology very help I’m sure those test would have been too. I just wish I knew about it earlier	Great presentation, very good in getting me up to speed again
Very useful as an advanced standing student for both my subjects: pharmacology and understanding disease concepts. The website gave me insight as to the content and concepts which would be discussed and studied throughout the semester	I feel like I’m ready to study now! Thanks!
	Any other comments: Negative comments
	Did not attend (review of Anatomy and Physiology and Microbiology) as email notice was given too soon prior to session. Will however follow up online through the Blackboard site
	(This orientation could be improved) by breaking it in parts. It (was) too much for one day

### Feedback on the “Workshop” part of the intervention; attendance and questionnaires

**Fig. 1:**
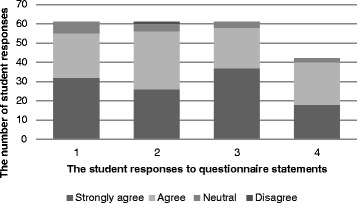
The student responses following evaluation of the Workshop presentations. The students were responding to the following questionnaire statements: 1 The library information session was informative and valuable. 2 The active learning session was informative and valuable. 3 The review of anatomy and physiology was informative and valuable. 4 The review of microbiology was informative and valuable

In 2011, 100 (45 %) of the accelerated students attended the workshop. However, only 61 responses to the questionnaire were obtained, as insufficient questionnaires were available, due to a greater than expected attendance at the workshop. On the 61 workshop questionnaires, most students “strongly agreed” or “agreed” the information they received was informative and valuable, and the talks on the “library resources” and “active learning” were useful (Fig. 1). The review lectures on anatomy and physiology and microbiology were also well received (Fig. 1). Although, the questionnaire allowed for student to add ‘any other comments’, very few students made any, and most were positive (Table 5).

In 2012, only 29 (13 %) accelerated students attended the workshop and all completed the questionnaire. The results were very similar in 2012 to 2011, and are not shown.

### Use of the community website on Blackboard

In 2011, 30 students accessed the community website. In 2012, the number of students who accessed the site increased to 152.

### After the intervention; student withdrawal and failure rates

After the introduction of the intervention in 2011, the withdrawal rates of accelerated students were reduced, and were no longer significantly different from those of the traditional students (Table 3). Subgroup analysis of the accelerated students showed that no international students withdrew in 2011-2012 (Table 4), and the withdrawal rates for the domestic non-graduates were no longer significantly different from those of the traditional students (Table 3).

After the intervention, only 4.9 % and 5.0 % of the accelerated students failed in 2011 and 2012, which was not significantly different from the rate for the traditional students, 6 % and 4 %, respectively. Subgroup analysis showed no international or domestic graduate students failed in 2011 or 2012 (Table 4). The failure rates for the domestic non-graduates were 5.8 % and 6.7 % in 2011 and 2012, respectively and these rates were not significantly different to those of the traditional students (Table 3).

### Percentage marks of retained students

**Fig. 2:**
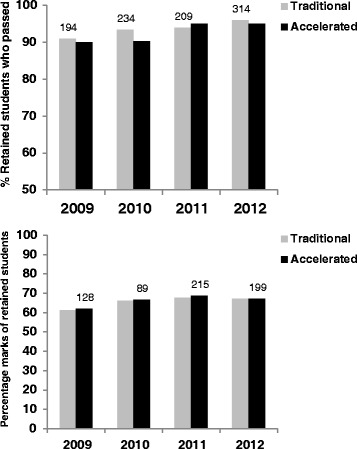
Retained traditional and accelerated students: top; percentage that passed; bottom; percent marks of students that passed. The numbers in each group are given above the column for the traditional students in the top graph and for the accelerated students in the bottom graph

The percentage marks of the students who passed were not significantly different, as determined using student’s unpaired *t*-test, between the traditional and accelerated students before and after the intervention (Fig. 2).

## Discussion

### Age

At our institution, the accelerated nursing students are on average five years older than the traditional nursing students. This is in line with a study of graduate entry and traditional nursing students at the University of Western Sydney, which showed that the graduates were about four years older than the traditional students [16]. This is as expected, as many of the traditional students have come to university after completing secondary school, whereas the accelerated students are university graduates or had non-university tertiary qualifications, or equivalent experience. To our knowledge, there is no research into whether age alone has an influence on outcomes in nursing education. In our study, it seems unlikely that age alone had an influence on outcomes, as the older graduate accelerated students (both international and domestic graduates) had similar or better outcomes (withdrawal, failure and success rates) than the younger traditional students. The domestic graduates and non-graduates have similar ages in 3 of the 4 years studied, and in the other year, the non-graduates are younger than the graduates. However, despite being similar in age, the non-graduates consistently have poorer outcomes than the graduates; this suggests the poorer outcomes are not related to age.

### Before the intervention, student withdrawal and failure rates

The study of the accelerated students in the pharmacology course showed that the international students and domestic graduate students had low withdrawal and failure rates. In contrast, the domestic students with non-university qualification or equivalent experience had higher withdrawal and failure rates. Our low withdrawal and failure rates observed for international students are in accordance with previous findings [8]. Previous studies have also shown that non-nursing graduates completing accelerated nursing programs, including in Australia, did as well or better than students completing traditional nursing programs [6, 7]. In our study, the domestic graduate students have similar withdrawal and failure rates to traditional students.

The reasons for the low withdrawal and failure rates of domestic graduates and international students may include development of knowledge and skills though a prior university experience. Additionally students in the pharmacology course are provided with an eBook ‘Pharmacology in One Semester’ [17] rather than a standard textbook. This eBook is designed for an introductory course in pharmacology, is in plain English, tailored to the course, and is about 200 pages long, which is less than a fifth of the size of the standard textbook [17]. This eBook was prepared and internally reviewed by pharmacology academics from 5 Australian universities, and is available on a Google website [17]. In the yearly evaluations of the pharmacology course at our university, most students consider this eBook to be the best component of the course [18]. The eBook has been available since the introduction of the pharmacology course, and thus we do not know whether it has an effect on withdrawal and failure rates. However, it is possible that the eBook may contribute to the overall low withdrawal and failure rates in the course, including the very low withdrawal and failure rates of domestic graduates and international students. It is also possible the domestic non-graduates may have had even higher withdrawal and failure rates without access to this eBook.

Another possible reason for the high retention and success rates of these students in pharmacology is that the concomitant bioscience courses are well suited to their needs, and provides a good foundation for the pharmacology course. Additionally, there are also high numbers of these students in the nursing program and anecdotally they are supportive of each other.

In contrast, the withdrawal and failure rates of accelerated domestic nursing students with non-university qualifications or equivalent experience are higher at our university than for the traditional students. Our university teaches nursing students on two campuses, and the present study relates to the larger of these two campuses. However, the results are consistent at both campuses, as it has previously shown that the withdrawal rates of the accelerated students with non-university qualifications are higher than for the traditional students at our smaller campus [19]. There are two other studies from Australian universities suggesting higher withdrawal and failure rates of accelerated students with non-university qualifications or equivalent experience [20, 21]. These findings concur with the notion identified by Ralph et al (2013) that diploma nurses are underprepared for university [22]. At present, the Australian government is committed to a seamless transition for these non-university nursing graduates (diploma students) from TAFE to the second level of a nursing degree [9]. Thus, the universities in Australia have to meet the challenge of these diploma students without altering their entry requirements. At our university, we have devised an intervention that occurs just prior to, and at the start of, their first semester to help combat any under-preparedness of accelerated students, including diploma students.

### Intervention

The main part of our study was to use an intervention in an attempt to improve the retention and success of the accelerated students in nursing. Our intervention was based on the study by Yorke & Thomas (2003) of six UK universities, which were performing above the average for completion rates for mature age entrants and for students with low socioeconomic backgrounds; the authors identified factors that could have a positive outcome on retention [11]. The factors addressed in this study were (i) provision of formative assessment in the early phase of the program (ii) provision of an institutional climate supportive in various ways of students’ development, and (iii) provision of support leading up to the critically important first year of study [11]. Although the graduate accelerated students at our university campus do not come from a low SES, the non-graduate diploma students are predominantly from TAFE, which draws from a low SES background. Thus, we considered that the factors identified by Yorke & Thomas may serve as a useful starting point for the development of strategies to support accelerated student cohorts.

The intervention created a supportive climate for the accelerated nursing students’ first experience of university in the provision of a workshop, resource lectures and a community website. Both the workshop and the resource lectures constituted extra support at the start of the first year of study and allowed for student-staff interaction. “Getting Started” was a formative assessment for use prior to or at the start of semester. The “O Week Workshop” and “Resource lectures” were perceived, by most of the responding accelerated students, as valuable and informative. The feedback from the students indicated that these provisions were successful in supporting accelerated students.

The student responses to the end-of-semester questionnaire on “Getting Started” were positive; however it was difficult to infer the value of “Getting Started” from this questionnaire due to the small number of respondents. Sending the follow-up questionnaire to students earlier in the semester may have resulted in an increased response rate to our end-of-semester questionnaire. Additionally, the uses and advantages of the community website could have been better advertised to the student cohort, although it is difficult to get access to students until just before ‘O week’.

After the intervention, the withdrawal and failure rates of the accelerated domestic non-university students in the pharmacology course were reduced, and were similar to the rates of the traditional students after the introduction of the intervention in 2011 and 2012. Thus, our intervention is associated with a reduction in withdrawal and failure rates, and may contribute to the reduction. One of the reasons the intervention may have reduced withdrawal and failure rates is linked to the pharmacology second year level course that presupposes certain anatomy and physiology knowledge. However, prior to the intervention, many of these students may have had limited prior knowledge of these topics, despite being awarded academic credit for previous learning or workplace or life experience, which may have accounted for higher withdrawal and failure rates in 2009 and 2010. The “Resource Lectures” in the intervention may have helped plug this gap, and contributed to the improved success of the accelerated domestic non-university students.

The nursing students (traditional and accelerated) undertake the same pharmacology course, with other allied health professionals, in very large classes (between 650 and 820 students over the four year study period). Thus, there is little opportunity for interaction between faculty and the accelerated nursing student cohort, outside of the support given in the intervention, and therefore it is unlikely faculty support during the semester contributed to the improved retention and success of the domestic non-university accelerated students The authors are not aware of any changes to the nursing admissions or program, during this study that would have improved the retention of these accelerated students. The traditional students in the pharmacology course, who had already completed a first year course of bioscience, provide a non-equivalent comparison group for our study. The withdrawal, failure and pass rates, and marks for these traditional students were similar over the 2009 to 2012 period, which indicates that little was changing in the pharmacology course over this time.

There are several limitations to our study. Unfortunately at our university, we are only able to access class lists and to contact students, in the week prior to semester. Thus, the students had very short notice to attend the workshop. Consequently the number of students attending the workshop in 2011 was very low (13 %). However, although we do make these activities available on the community website via recordings and lecture notes, we have not been able to monitor accelerated students access to date; this constitutes a major limitation which we plan to address in the future.

Another limitation is that although we report the gender of both the accelerated and traditional students in the classes as being predominantly female, we were unable to determine whether gender was a significant factor in the withdrawal and failure rates for the domestic non-university qualification/equivalent experience group, as the absolute number of male and female students’ withdrawal and failure rates were low.

Another limitation to our study is that we do not know how many of our international graduates have English as a second language. This information is not available for up to 83 % of students in some cohorts, as the provision of this information by the students is voluntary, not mandatory. As teachers in nursing, we are aware the international students are predominantly English as second language students, and the domestic students are predominantly English speaking. However, we cannot quantify this, and determine whether this is a factor in withdrawal and failure rates, but it seems unlikely, as the international students have low withdrawal and good success in our course.

Although this intervention strategy has only been conducted at one university, similar findings with regard to accelerated domestic students with non-university qualifications or equivalent experience, have been found at both campuses [19,23]. We would like to test our intervention at other universities enrolling students with non-university qualifications or equivalent experience, and this is in line with the suggestion of Kuh et al (2007), who reported that research is needed to verify effective approaches that foster success of different groups of students at different types of institution [15]. Our long term goal is to provide an intervention, which can be used throughout Australia, to reduce the withdrawal and failure rates of accelerated domestic students with non-university qualifications or equivalent experience.

## Conclusions

Accelerated domestic students with non-university qualifications or equivalent experience have higher withdrawal and failure rates in a pharmacology course than accelerated international students or domestic graduates or traditional nursing students. Following the intervention introduced by the authors at the start of 2011, the withdrawal and failure rates of the accelerated and traditional students were no longer significantly different. Additionally, the favourable student responses, to the “O Week Workshop” and “Resource lectures”, indicated that the accelerated students in this course felt supported. Therefore, the intervention employed in this study, namely the community website, formative assessment, the workshop and anatomy, physiology and microbiology review lectures seem to have supported and increased the retention rates of these accelerated students. Other universities enrolling accelerated students could consider using this intervention to improve the retention and success of their accelerated students.
